# Supplementary material for: Integrated Analysis of the Lung Microbiome and Metabolome Reveals Associations Between Amino Acid Metabolism and Pulmonary Fibrosis in a Bleomycin-Induced Mouse Model
Source: Int J Mol Sci. 2026 Jun 30;27(13):5895. doi: 10.3390/ijms27135895 (PMC13362081; doi:10.3390/ijms27135895)
Supplement: Supplementary file 1 [file ijms-27-05895-s001.zip › result/5.MetKeggEnrichment/B_28d.vs.C_28d/B_28d.vs.C_28d_all_KEGG_classification.pdf]

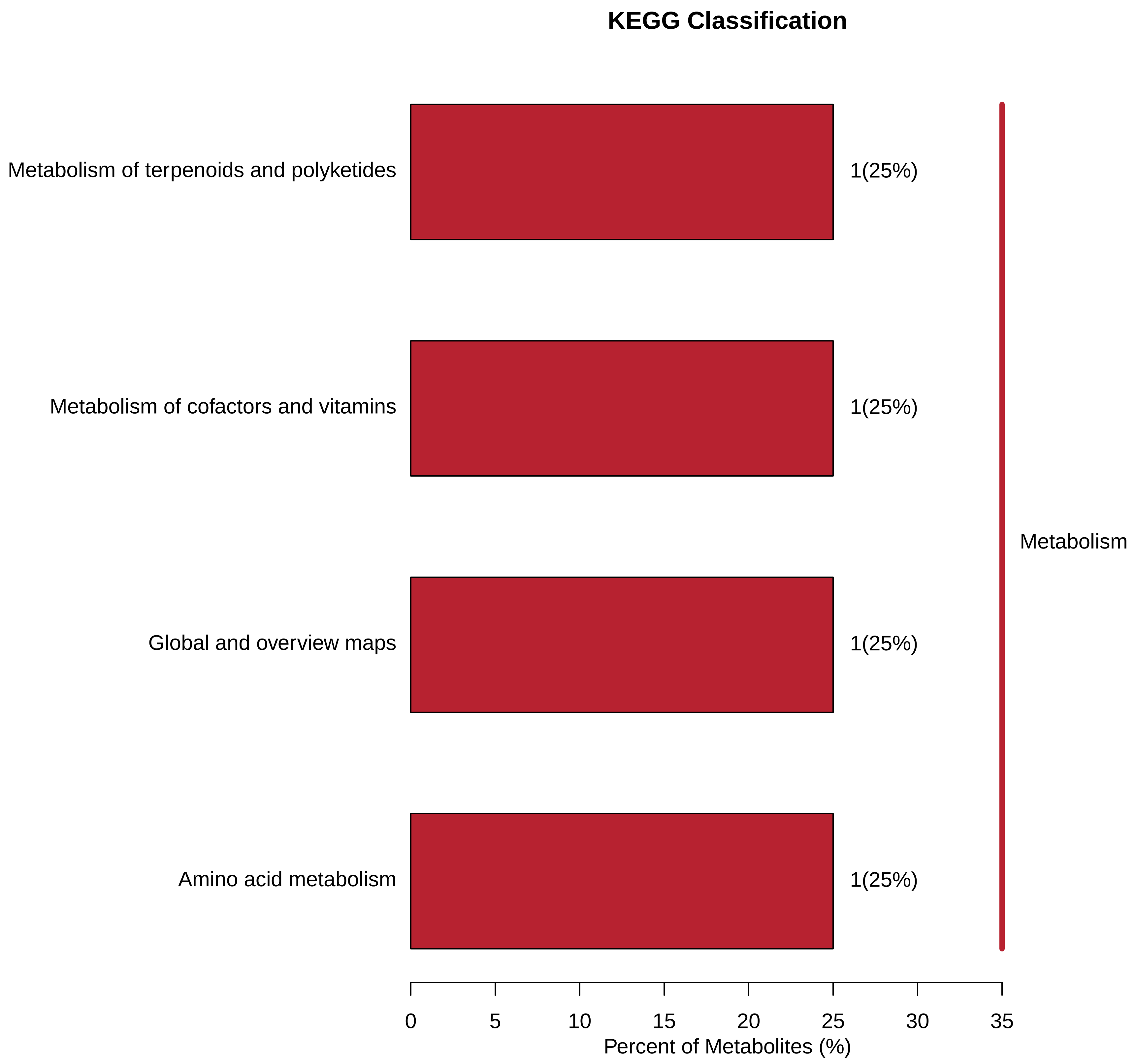
KEGG Classification
Metabolism of terpenoids and polyketides
Metabolism of cofactors and vitamins
Global and overview maps
Amino acid metabolism
1(25%)
1(25%)
1(25%)
1(25%)
Metabolism
0
5
10
15
20
25
30
35
Percent of Metabolites (%)
